# [2,2′-Bithiophene]-4,4′-dicarboxamide: a novel building block for semiconducting polymers†

**DOI:** 10.1039/c9ra06909g

**Published:** 2019-09-25

**Authors:** Xiaocheng Zhou, Zhifang Zhang, Arthur D. Hendsbee, Jenner H. L. Ngai, Pankaj Kumar, Shuyang Ye, Dwight S. Seferos, Yuning Li

**Affiliations:** Department of Chemical Engineering/Waterloo Institute for Nanotechnology (WIN), University of Waterloo 200 University Ave West Waterloo ON N2L 3G1 Canada yuning.li@uwaterloo.ca; Lash Miller Chemical Laboratories, Department of Chemistry, University of Toronto 80 St. George Street Toronto Ontario M5S 3H6 Canada

## Abstract

A novel electron deficient building block [2,2′-bithiophene]-4,4′-dicarboxamide (BTDCA) was designed to lower the highest occupied molecular orbital (HOMO) energy level of polythiophenes in order to achieve a higher open circuit voltage (*V*_oc_) and thus a higher power conversion efficiency in polymer solar cells (PSCs). BTDCA dibromo monomers were conveniently synthesized in four steps, and were used to prepare three thiophene-based D-A polymers, P(BTDCA66-BT) (66BT), P(BTDCA44-BT) (44BT) and P(BTDCA44-TT) (44TT). All the polymers exhibited unipolar hole transport properties, exhibiting mobilities in the range of ∼10^−4^ to 10^−2^ cm^2^ V^−1^ s^−1^ with the highest hole mobility of up to 1.43 × 10^−2^ cm^2^ V^−1^ s^−1^ achieved for 44BT in bottom-gate bottom-contact organic thin film transistors (OTFTs). In PSCs, these polymers achieved high *V*_oc_'s of 0.81–0.87 V when PCBM or ITIC was used as acceptor. When 44TT was used as donor and ITIC was used as acceptor, a power conversion efficiency (PCE) of up to 4.5% was obtained, a significant improvement when compared with the poly(3-hexylthiophene) (P3HT):ITIC devices, which showed the highest PCE of merely 0.92%.

## Abstract 2

A new electron acceptor building block, [2,2′-bithiophene]-4,4′-dicarboxamide, is synthesized and used to develop donor polymers for organic solar cells.

## Graphical abstract

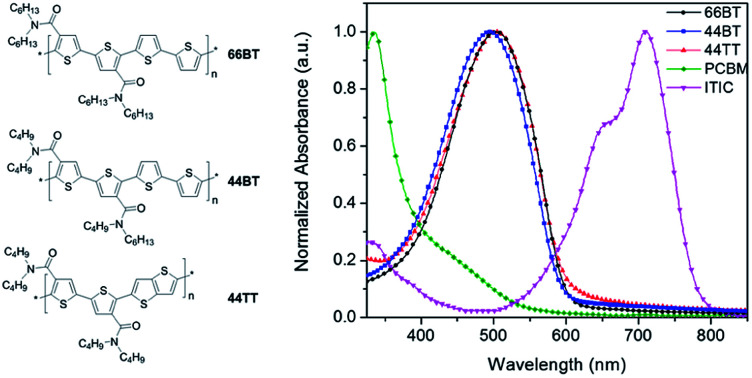

## Introduction

Polymer solar cells (PSCs) have been of considerable academic and industrial interest, owing to their light weight, solution processability, and mechanical flexibility.^1–17^ The highest PCEs have reached 16.5% and 17.3% for single-junction^4^ and tandem^5^ PSCs, respectively, demonstrating a closer step to commercialization of PSCs. Among a number of obstacles to the commercialization of PSCs, the cost of organic semiconductor materials including the polymer donors is still inhibitive. All the high-performing polymer donors reported so far require very complicated synthesis or have high synthetic complexity (SC),^18^ a parameter that can be directly correlated to the cost of the polymer donor. Regioregular head-to-tail poly(3-hexylthiophene) (P3HT) can be synthesized with the simplest synthetic route and thus has the lowest SC among all reported donor polymers. P3HT has been extensively investigated as a donor to couple with the fullerene-based acceptor, [6,6]-phenyl-C_61_-butyric acid methyl ester (PCBM), in the past years.^19–22^ However, the PCE of the P3HT:PCBM based solar cells remains low at *ca.* 3–4%.^19,22,23^ P3HT also performs poorly when the currently best performing small molecule non-fullerene acceptors, *e.g.*, ITIC^24^ as well as polymer acceptors, *e.g.*, PNDI(2OD)2T,^25^ are used. The poor solar cell performance of P3HT based PSCs is mainly due to its unnecessarily high HOMO (highest occupied molecular orbital) energy level, *ca.* −5.0 eV,^26–28^ which results in lower open circuit voltages (*V*_oc_'s).^29,30^ To pursue higher *V*_oc_'s, researchers have designed and synthesized various polythiophene derivatives by tuning the polymer backbone to achieve deeper HOMO energy levels.^31–35^ Alternatively, other types of non-fullerene acceptors, such as IDTBR derivatives with higher LUMO energies, have been developed to match with the HOMO energy of P3HT, resulting higher *V*_oc_'s and PCE's.^36^

Herein, we report a new strategy to lower the HOMO energy level of polythiophenes by simply anchoring amide functional groups to bithiophene to form a new building block, [2,2′-bithiophene]-4,4′-dicarboxamide (BTDCA). Three new thiophene-based polymers incorporating this building block have demonstrated deeper HOMO energy levels of −5.3 eV to −5.4 eV, resulting in significant increases in *V*_oc_ (0.87 V) compared with P3HT (*V*_oc_ = *ca.* 0.52 V) when ITIC was used as the acceptor in PSCs. A PCE of up to 4.5% was achieved by one of the BTDCA based polymers.

## Results and discussion

### Design and synthesis of BTDCA-based polymers

**Fig. 1:**
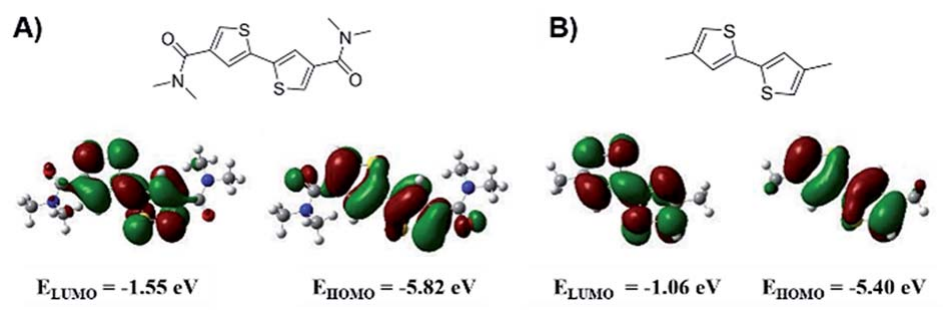
DFT calculation results for (A) BTDCA-Me and (B) BT-Me.

The work commenced by conducting computer simulations on two model compounds, *N*^4^,*N*^4^,*N*^4′^,*N*^4′^-tetramethyl-[2,2′-bithiophene]-4,4′-dicarboxamide (BTDCA-Me) and its methyl substituted analogue 4,4′-dimethyl-2,2′-bithiophene (BT-Me) shown in Fig. 1 using a density functional theory (DFT).

The simulated results show that the electrons in the HOMO and LUMO of BTDCA-Me are quite evenly delocalized across the two thiophene rings, which can be beneficial for charge carrier transport.^37^ Importantly, by attaching amide electron withdrawing groups, the HOMO and LUMO energies of BTDCA-Me are significantly lowered to −5.82 eV and −1.55 eV, respectively, compared with BT-Me, which has HOMO and LUMO energies of −5.40 eV and −1.06 eV, respectively. These results suggest that by incorporation of the BTDCA building block in the polythiophene backbone, the resulting polymer would be able to achieve a much lower HOMO energy and thus a higher *V*_oc_ in PSCs with respect to P3HT.

**Scheme 1:**
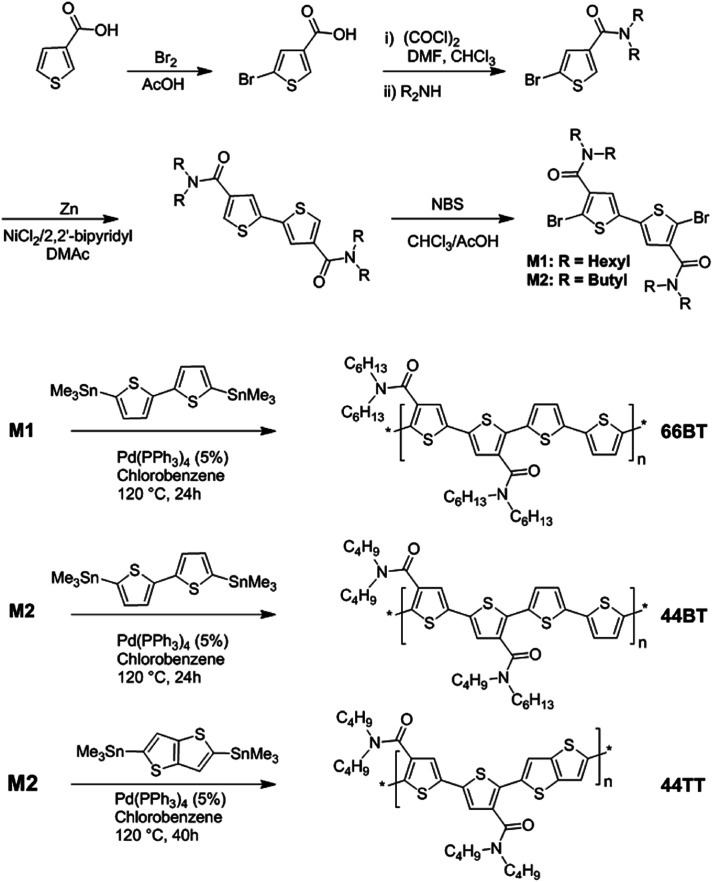
Synthesis of BTDCA monomers and polymers 66BT, 44BT, and 44TT.

Two dibromo BTDCA monomers, M1 and M2, with dihexyl and dibutyl side chains were synthesized in four steps starting from commercially available 3-thiophenecarboxylic acid following the procedure outlined in Scheme 1. BTDCA-based polymers, 44BT, 66BT and 44TT, were then synthesized *via* Stille coupling polymerization of M1 and M2 with 5,5′-bis(trimethylstannyl)-2,2′-bithiophene or 2,5-bis(trimethylstannyl)-thieno[3,2-*b*] thiophene, respectively, using Pd(PPh_3_)_4_ as a catalyst in chlorobenzene as solvent at 120 °C for 24 h. The crude polymers were precipitated from methanol and then subjected to Soxhlet extraction with acetone, hexane and chloroform. The chloroform fraction was dried under vacuum to afford 66BT, 44BT and 44TT as shiny dark purple solids with yields of 80%, 78% and 78%, respectively. The number average molecular weight (*M*_n_) of 66BT was 23.4 kDa with a dispersity (*Đ*) of 2.5, which was determined by HT-GPC using 1,2,4-trichlorobenzene as eluent at 140 °C. Although 44BT and 44TT are soluble in warm 1,2,4-trichlorobenzene, their molecular weights could not be determined by HT-GPC probably due to the strong aggregation of their polymer chains in solution. Instead a MALDI-TOF mass spectrometer was utilized to obtain the *M*_n_/*Đ* of 6.5 kDa/1.1 for 44BT and 7.7 kDa/1.1 for 44TT by using a DCTB matrix at a matrix-to-polymer ratio of 2500 : 1.

The thermal stability of the polymers was characterized by thermogravimetric analysis (TGA). A 2% weight loss was observed at 430 °C, 441 °C and 398 °C for 66BT, 44BT and 44TT, respectively, indicating the good thermal stability of these polymers (ESI†). We also conducted differential scanning calorimetry (DSC) on 66BT, 44BT and 44TT. However, no noticeable endo- or exothermic transitions were observed in the temperature range between −20 °C and 300 °C.

### Optoelectronic properties

**Fig. 2:**
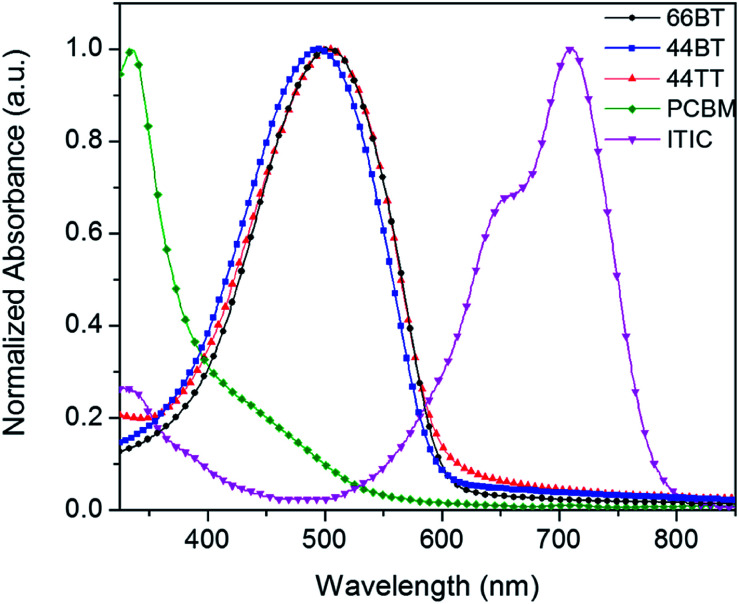
UV-visible spectra of polymers 66BT, 44BT, 44TT, PCBM, and ITIC films.

The photophysical characteristics of the polymers were investigated by measuring the UV-Vis-NIR absorption spectra of polymers in solution (see the ESI†) and as thin films (Fig. 2 and Table 1). The polymers exhibited single broad absorption peaks in the wavelength range of >350 nm in both solution and solid state, which is typical for thiophene-based polymers.^38^ In solution, the wavelengths at maximum absorption (*λ*_max_) of 66BT, 44BT and 44TT were found to be 487, 487 and 483 nm, respectively. The absorption spectra of three polymers in thin films are red-shifted to 503, 496, and 505 nm, respectively, which is attributable to aggregation effects such as planarization and *J*-aggregation in the solid state.^39^ The optical bandgaps (*E*^opt^_g_) of the polymer films are estimated to be 2.06 eV, 2.08 eV and 2.05 eV for 66BT, 44BT and 44TT, respectively, from the absorption onsets. As clearly seen in Fig. 2, all three polymers have complementary absorption profiles with that of the acceptor ITIC, covering the absorption region of *ca.* 350–800 nm in the solar spectrum.

**Table 1:** Physical properties of 66BT, 44BT and 44TT

Polymer	*λ*_max_ a (nm)	*λ*_max_ b (nm)	*E* ^opt^_g_ c (eV)	*E*_HOMO_ c (eV)	*E*_LUMO_ d (eV)
66BT	487	503	2.06	−5.30	−3.24
44BT	487	496	2.08	−5.30	−3.22
44TT	483	505	2.05	−5.40	−3.35
P3HT	451	505	1.85	−5.05	−3.20
ITIC	676	707	1.70	−5.75	−4.05

a Determined using the absorption profiles of solution samples in chloroform.

b Determined from the as-cast thin films.

c Determined using cyclic voltammetry curves of the thin films.

d Determined using a combination of the optical band-gap and the HOMO energy levels obtained from CV measurements of thin film samples.

**Fig. 3:**
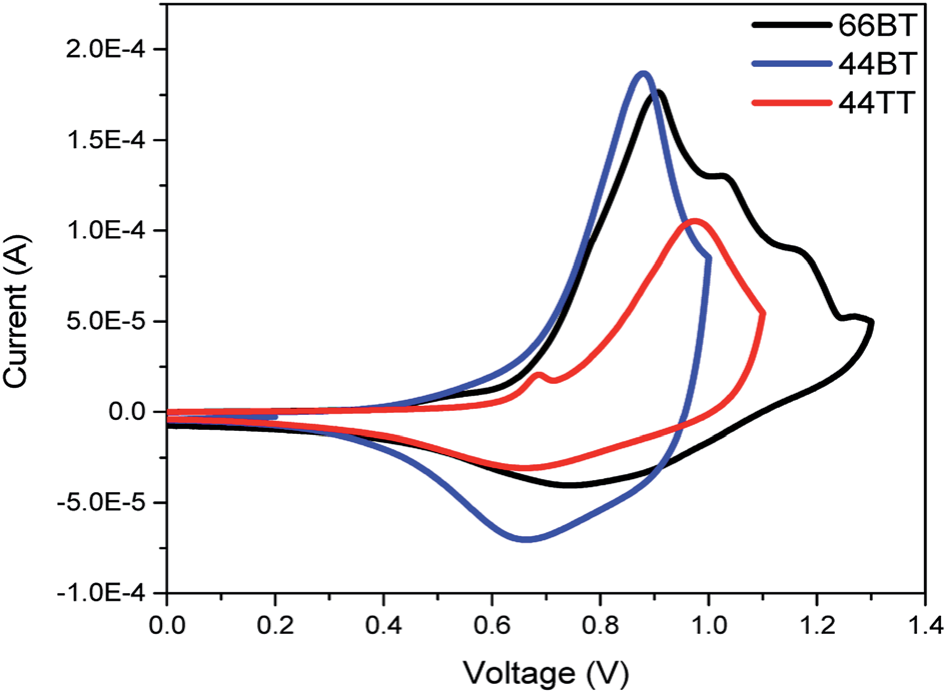
Cyclic voltammograms of polymer films acetonitrile solution with tetrabutylammonium fluoride (0.1 M) as an electrolyte.

Cyclic voltammetry (CV) was then used to measure the electron affinity (EA) and ionization potential (IP) of the polymers, which can be used as close estimates for the LUMO and HOMO energy levels, *i.e.*, *E*_LUMO_ ≈ −*E*_A_ and *E*_HOMO_ ≈ −IP, respectively.^40^ As shown in Fig. 3, the cyclic voltammograms of the three polymers exhibited strong oxidation processes. Based on the onsets of oxidation, the HOMO energy levels were estimated to be −5.30 eV for both 66BT and 44BT and −5.40 eV for 44TT eV, using Fc/Fc^+^ as an internal reference. These HOMO energy levels are notably deeper than that (−5.05 eV) of P3HT measured under same conditions. The measured HOMO values agree with the simulated results that the BTDCA building block helps bring down the HOMO energy levels, a desirable characteristic for achieving a higher *V*_oc_ in PSCs using these polymers as donors.

Due to the absence of reduction peaks, the LUMO energy levels were calculated by adding the optical bandgaps to the HOMO energy levels obtained from CV. Using this method, the LUMO energy levels of the polymers were found to be −3.24 eV, −3.22 eV and −3.35 eV for 66BT, 44BT and 44TT, respectively.

### Organic thin-film transistor (OTFT) performance

The charge transport properties of three polymers were characterized in bottom-gate bottom-contact (BGBC) OTFTs, which were fabricated on dodecyltrichlorosilane (DDTS) modified SiO_2_/Si wafers with Au pairs as the source and drain contacts. The polymer films were deposited by spin-coating polymer solutions on the substrates in ambient air and then the devices were sequentially annealed and characterized in an argon-filled glovebox. All devices showed unipolar p-type charge transport characteristics (Table 2). Among three polymers, 44BT exhibited the highest hole mobility of 1.4 × 10^−2^ cm^2^ V^−1^ s^−1^ at an annealing temperature of 200 °C, followed by 66BT that showed the maximum mobility of 2.0 × 10^−3^ cm^2^ V^−1^ s^−1^, while 44TT showed the poorest charge transport performance with a highest value of 4.5 × 10^−4^ cm^2^ V^−1^ s^−1^ achieved at the annealing temperature of 200 °C. The mobility trend can be explained by their different degree of crystallinity (*vide infra*). Nonetheless, the mobility values of these polymers fall in the range of between *ca.* 10^−4^ cm^2^ V^−1^ s^−1^ and *ca.* 10^−2^ cm^2^ V^−1^ s^−1^ at annealing temperatures of 50 °C to 150 °C, suggesting that they may be suitable as polymer donors for PSCs.

**Table 2:** OTFT device data for 66BT, 44BT and 44TT

Polymer	Annealing temperature (°C)	*μ*_sat_ (cm^2^ V^−1^ s^−1^)	*V*_th_ (V)	*I*_ON/OFF_
66BT	50	2.6 × 10^−4^	−52	10^3^
	100	1.1 × 10^−3^	−56	10^3^
	150	2.0 × 10^−3^	−41	10^3^
	200	3.5 × 10^−4^	−56	10^4^
44BT	50	3.3 × 10^−4^	−37	10^3^
	100	6.7 × 10^−3^	−38	10^4^
	150	1.3 × 10^−2^	−38	10^4^
	200	1.4 × 10^−2^	−37	10^5^
44TT	50	9.4 × 10^−5^	−26	10^3^
	100	9.4 × 10^−5^	−33	10^3^
	150	1.0 × 10^−4^	−39	10^3^
	200	4.5 × 10^−4^	−44	10^3^

### X-ray diffraction (XRD) analysis

**Fig. 4:**
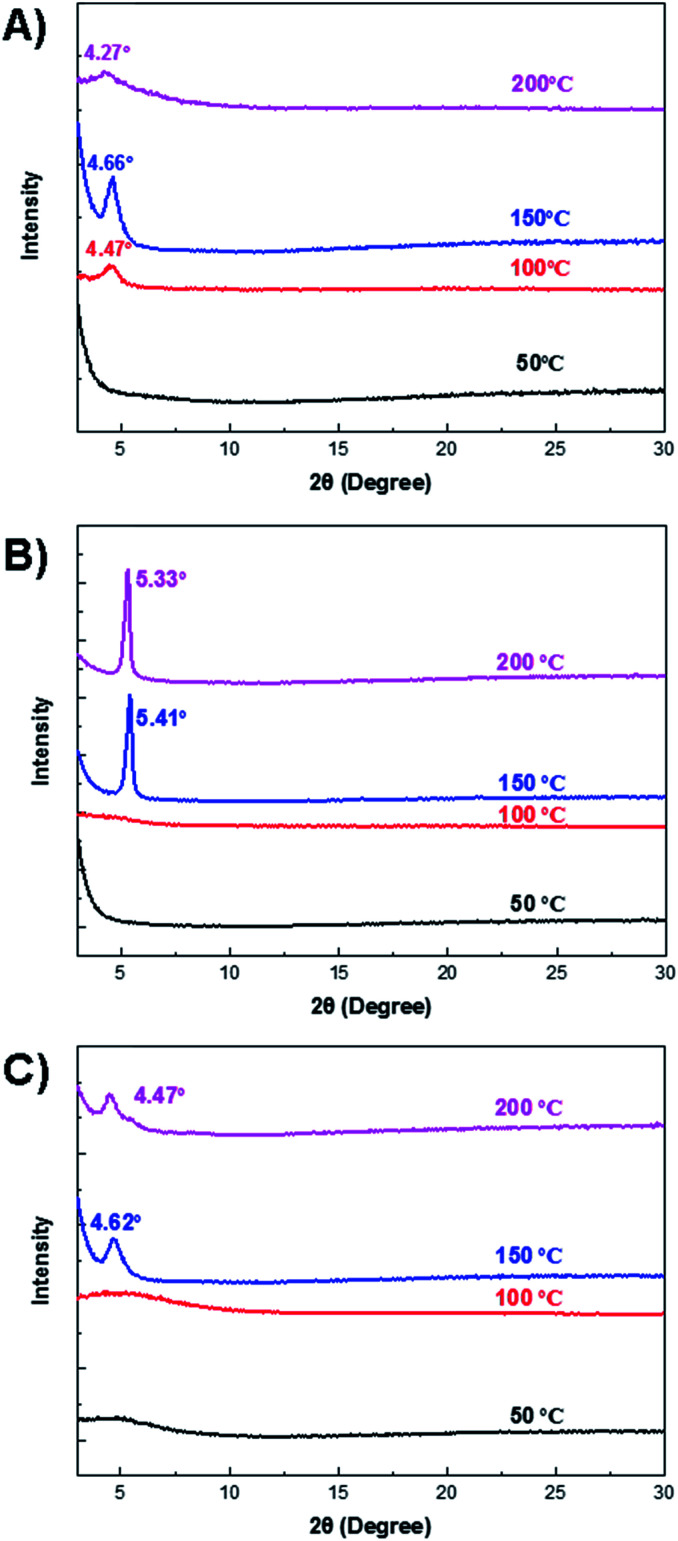
X-ray diffraction patterns of polymer films cast on silicon substrates.

The crystallinity of the polymer thin films was characterized by the XRD measurement (Fig. 4). For the 66BT thin film annealed at 50 °C, no distinct diffraction peak was observed, indicating amorphous chain packing of this polymer. After annealing at 100 °C, a clear diffraction peak appeared at 2*θ* = 4.47°, which could be assigned to the (100) diffraction peak, corresponding to an interlayer lamellar *d*-spacing of 1.97 nm. At the annealing temperature of 150 °C, the (100) peak significantly intensified and shifted to 2*θ* = 4.66°, which corresponds to a shortened *d*-spacing of 1.90 nm. The highest crystallinity and tightened interchain packing account for the best hole mobility of 66BT in OTFTs obtained at this annealing temperature. At the annealing temperature of 200 °C, the (100) peak weakened and shifted to 2*θ* = 4.66° (*d* = 2.07 nm), indicating the reduced crystallinity and widened interlamellar distance, which can explain the drop in mobility at this annealing temperature. Compared to 66BT, 44BT exhibited more intense diffraction peaks at 2*θ* = 5.41° and 5.33° for the thin films annealed at 150 °C and 200 °C, corresponding to an interlayer lamellar *d*-spacing of 1.63 nm and 1.66 nm, respectively. The thin film of 44TT annealed at 50 °C and 100 °C showed broad diffraction peaks, indicating less ordered chain packing of this polymer at these annealing temperatures. After annealing at 150 °C, a diffraction peak appeared at 2*θ* = 4.62° (*d* = 1.91 nm), which is much weaker than those of 66BT and 44BT annealed at the same temperature. Similarly, when the annealing temperature increased to 200 °C, the peak shifted to a smaller 2*θ* of 4.47°, which corresponds to an increased interlayer lamellar *d*-spacing of 1.98 nm. It is well known that the crystallinity of polymer semiconductors plays a dominant role in the charge transport,^41,42^ which can be observed in this series of polymers, where the highest mobilities are obtained for the most crystalline polymer 44BT films.

### Photovoltaic properties

The polymers were used as the donor component in polymer donor:small molecule acceptor PSC devices to investigate their photovoltaic properties. The device configuration used in this work is ITO/PEDOT:PSS/polymer donor:acceptor/LiF/Al.

**Fig. 5:**
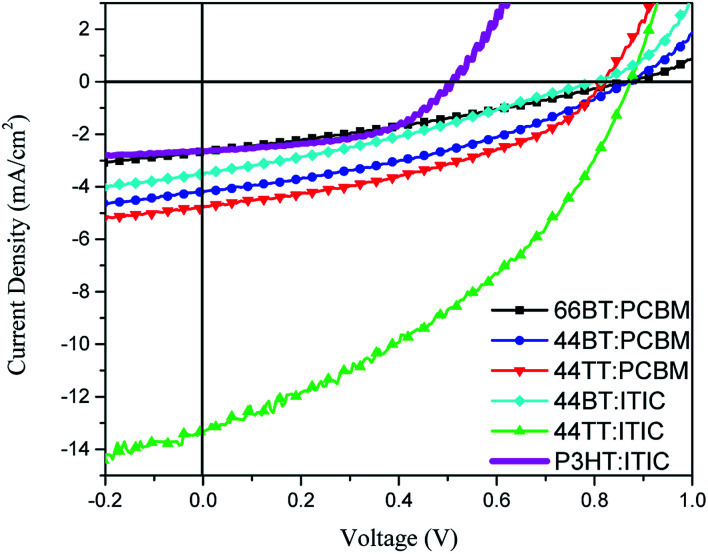
*J*–*V* curves for 66BT:PCBM, 44BT:PCBM, 44TT:PCBM, 44BT:ITIC, 44TT:ITIC and P3HT:ITIC solar cells under AM 1.5G illumination (100 mW cm^−2^).

Initially, PCBM was used as the acceptor material and different solvents, chlorobenzene (CB) and dichlorobenzene (DCB), were used to fabricate the solar cells for all three polymers, 66BT, 44BT and 44TT. The best results for each polymer are shown in Fig. 5 and summarized in Table 3 (a full account of the data can be found in Table S2 in the ESI†). It was found that 44BT and 44TT bearing the shorter butyl chains on the amide functional groups achieved PCEs of 1.31% and 1.59%, respectively, which are higher than that (0.72%) of the devices of 66BT bearing the longer hexyl chains. The *V*_oc_'s are 0.87 V, 0.87 V, and 0.82 V for 66BT, 44BT and 44TT based devices, respectively, much larger than that (*ca.* 0.6 V) of the P3HT:PCBM devices.^19–22^ This is a result of the higher HOMO energy levels of these BTDCA based polymers, originating from the electron withdrawing amide groups.

**Table 3:** Summary of PSC performance

Active layer	D/A ratio	Solvent	*J*_sc_ (mA cm^−2^)	*V*_oc_ (V)	FF	PCE%
66BT:PCBM	1 : 1	CB	2.7	0.87	0.31	0.72
44BT:PCBM	1 : 1	DCB	4.2	0.87	0.36	1.31
44TT:PCBM	1 : 1	CB	4.8	0.82	0.41	1.59
44BT:ITIC	1 : 1	DCB	3.5	0.81	0.30	0.85
44TT:ITIC	1 : 1	DCB	13.2	0.87	0.39	4.47
P3HT:ITIC	1 : 1	CF	3.33	0.52	0.53	0.92

Recently ITIC and its derivatives have become the most popular non-fullerene small molecule acceptors for PSCs since they have achieved very high PCE's.^43–47^ Compared to PCBM, which does not absorb much light above 400 nm, ITIC can harvest solar energy more efficiently, absorbing strongly in the region between 500 nm and 800 nm.^43^ As can be clearly seen in Fig. 2, the three polymers have quite complementary absorption profiles with that of ITIC and thus the PSCs using these polymers as donors and ITIC as the acceptor would cover the solar spectrum range from *ca.* 350 nm to 800 nm. In addition, the HOMO and LUMO energy levels of ITIC are −5.75 eV and −4.05 eV (Table 2), respectively, which match well with those of these BTDCA polymer donors to have sufficient energy offsets for exciton dissociation with quite small energy losses.

**Fig. 6:**
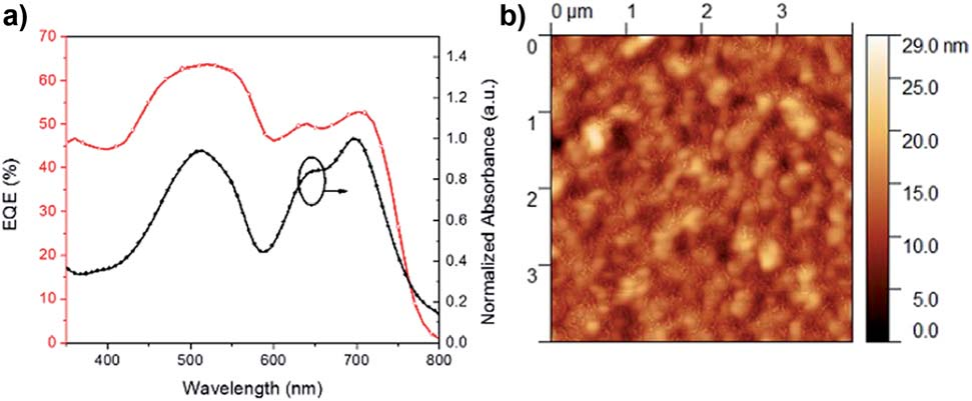
(a) EQE curves and absorbance of PSCs based on 44TT:ITIC and (b) the AFM image of surface morphology of the 44TT:ITIC film cast from DCB (RMS: 2.6 nm).

When ITIC was used to test the solar cell performance of polymers 44BT and 44TT, a much improved PCE% of 4.47% was achieved for the 44TT:ITIC devices. Compared with the 44TT:PCBM based devices, which showed a PCE of 0.85%, the efficiency improvement of the 44TT:ITIC devices is mainly due to the increased photocurrent generation (13.2 *vs.* 4.8 mA cm^−2^). As shown in Fig. 6, the EQE values based on 44TT:ITIC exceed 45% in the range of 600–800 nm where ITIC is mainly responsible for light harvesting. In addition, the high EQE values over 60% in the absorption region of 44BT (*ca.* 400–600 nm) indicate that this polymer donor converts photons to photocurrent more efficiently than ITIC. The *J*_sc_ calculated from EQE spectrum is 12.63 mA cm^−2^, which agrees well with the value obtained from the *J*–*V* measurement. The *V*_oc_ is also quite high at 0.87 V. Previously Qin *et al.* studied the P3HT:ITIC based PSCs, which only afforded PCE of 1.25%,^24^ while the best PCE we obtained for P3HT:ITIC system under our processing conditions is 0.92% (Table 1). Compared to the P3HT:ITIC devices, the 44TT:ITIC PSCs have much higher *J*_sc_ and *V*_oc_ values. However, the fill factor obtained for 44TT:ITIC device is only 0.39, which is much lower than the P3HT:ITIC device (0.57) and those of the state-of-the art PSC devices (up to 0.80).^7,11^ The relatively poor film morphology of 44TT:ITIC might account for the lower fill factor. The 44BT:ITIC based devices showed much lower performance with a maximum PCE of 0.85%, which is even lower than that of the 44BT:PCBM based devices. This is mainly due to the poorer solubility of 44BT, causing severe phase separation in the 44BT:ITIC blend films.

AFM was used to examine the morphology of best-performing 44TT:ITIC blend film (Fig. 6b). The blend film is comprised of grains with sizes of *ca.* 50 nm, which seem too large compared with the blend film morphology of some best-performing PSCs^48,49^ and might account for the rather low fill factors of PSCs. We found that thermal annealing or adding solvent additives (*e.g.*, 1,8-diiodooctane, DIO) worsened the film morphology of the 44BT or 44TT blend with ITIC and reduced the PCE. As discussed previously on the molecular weight results, 44BT and 44TT readily form aggregates in solution, which might account for the poor morphologies obtained for their blend films. Further study on improving the morphology by optimizing the fabrication conditions, *e.g.*, type of solvent, spin-coating rate, other solvent additive as well as the polymer structure, *e.g.*, the alkyl substituents and donor building blocks will be conducted.

## Conclusions

In this work, we designed and synthesized a new electron-deficient building block, [2,2′-bithiophene]-4,4′-dicarboxamide (BTDCA), by simple chemistry in four steps. Using BTDCA as the electron acceptor unit, we constructed three D-A conjugated polymers: 66BT, 44BT and 44TT, which exhibited deeper HOMO energy levels when compared with P3HT. Among three polymers, 44BT showed the best p-type semiconductor performance with a highest hole mobility of 1.4 × 10^−2^ cm^2^ V^−1^ s^−1^ in OTFTs. As donor in polymer solar cells, 44TT exhibited the best performance, achieving high *V*_oc_ of 0.87 V and PCE of up to 4.47% when ITIC was used as an acceptor, which are much higher than those (*V*_oc_ = 0.52 V and PCE = 0.92%) of the P3HT:ITIC devices. These preliminary results demonstrate that BTDCA is a promising electron deficient building block for constructing p-type copolymers for OTFTs and OSCs.

## Conflicts of interest

There are no conflicts to declare.

## Supplementary Material

RA-009-C9RA06909G-s001
