# Supplementary material for: [2,2′-Bithiophene]-4,4′-dicarboxamide: a novel building block for semiconducting polymers
Source: RSC Adv. 2019 Sep 25;9(52):30496–502. doi: 10.1039/c9ra06909g (PMC9072092; doi:10.1039/c9ra06909g)
Supplement: RA-009-C9RA06909G-s001 [file RA-009-C9RA06909G-s001.pdf]

This journal is © The Royal Society of Chemistry 2019

Electronic Supplementary Information
X. Zhou, et al.

[2,2'-Bithiophene]-4,4'-dicarboxamide: A novel building block for semiconducting polymers

1. Characterization

MALDI-TOF mass spectra were performed using a Bruker AutoFlex Speed MALDI-TOF mass spectrometer from a matrix of *trans*-2-[3-(4-*tert*-Butylphenyl)-2-methyl-2-propenylidene]malononitrile (DCTB) at a matrix : polymer ratio of 2500:1 casted from chloroform. Thermogravimetric analysis (TGA) was carried out on TA Instruments SDT 2960 at a scan rate of 10 °C min^{-1} under nitrogen. The UV-Vis-NIR absorption spectra of polymers were recorded on a Thermo Scientific model GENESYS™ 10S VIS spectrophotometer. Cyclic voltammetry (CV) data were obtained on a CHI600E electrochemical analyser using an Ag/AgCl reference electrode and two Pt disk electrodes as the working and counter electrodes in a 0.1 M tetrabutylammonium hexafluorophosphate solution in acetonitrile at a scan rate of 100 mV s^{-1}. Ferrocene was used as the reference, which has a HOMO energy of -4.8 eV. NMR data was recorded with a Bruker DPX 300 MHz spectrometer with chemical shifts relative to tetramethylsilane (TMS, 0 ppm). XRD measurement are carried out with a Bruker D8 Advance powder diffractometer with Cu Kα radiation (λ = 0.15406 nm) using standard Bragg-Brentano geometry. AFM images were taken on thin films with a Dimension 3100 scanning probe microscope.

2. Fabrication and Characterization of OTFT Devices

The hole mobility of the polymer was measured in OTFT devices with the bottom-gate-bottom-contact (BGBC) configuration. The OTFT device fabrication was carried out as follows.

First, the gold source and drain pairs were patterned on a heavily n-doped SiO_2/Si wafer with 300 nm thickness of SiO_2 by conventional photolithography and thermal deposition methods. Then, the small substrates that contain a set of transistors were cut from the large wafer and were placed into a beaker containing acetone and sonicated in an ultra-sonic bath for 20 min at room temperature. Subsequently, acetone was removed and 2-propanol (IPA) was added followed by ultrasonication for an additional 20 min. After sonication, the substrates were dried and treated

with air plasma for 2 min. Substrates were then immersed in ethanol, chloroform, a 10 mM solution of octadecanethiol in ethanol for 1 hour, and ethanol in a covered petri dish successively. After that, substrates were immersed in 100 mL DI Water in a petri dish and four drops of 1: 10: 10 (HNO_3: HCl: H_2O) were added. The substrates were kept for one min, taken out, and rinsed with deionized water, followed by drying with nitrogen gas and subsequently on a hot plate at 120 °C for 10 min. In the next step, the substrates were put in a solution of dodecyltrichlorosilane (DDTS) in toluene (3 v% DDTS in toluene) at room temperature for 20 min. The substrates were then rinsed with toluene and dried under a nitrogen flow. Then a polymer solution in chlorobenzene or dichlorobenzene (ca. 5 mg/mL) was spin-coated onto the substrate at 1000 rpm for 60 s to obtain a polymer film, which was subjected to thermal annealing at different temperatures for 20 min at each temperature in an argon filled glove box. All the OTFT devices have a channel length (L) of 30 µm and a channel width (W) of 1000 µm and were characterized in the same glove box using an Agilent B2912A Semiconductor Analyzer. The hole mobilities in the saturation regime were calculated according to the following equation:

$$I_{DS} = \frac{\mu C_i W}{2L}(V_G - V_T)^2$$

where I_{DS} is the drain-source current, μ is charge carrier mobility, C_i is the gate dielectric layer capacitance per unit area (~ 11.6 nF cm^{-2}), V_G is the gate voltage, V_T is the threshold voltage, *L* is the channel length (30 µm), and *W* is the channel width (1000 µm).

3. Fabrication and Characterization Polymer Solar Cells

All the polymer solar cells were fabricated in the following configuration ITO/PEDOT: PSS/Active layer/LiF/Al. The ITO substrates immersed in deionized water and acetone were sonicated in an ultrasonic bath for 20 min each at 40 °C. Then the substrates were taken out and cleaned by Q-tips with acetone. Substrates were then sonicated for 20 min at 40 °C in IPA, followed by the cleaning using Q-tips again. The substrates were dried in vacuum and then treated in an air plasma cleaner for 10 min. A ~40 nm thin layer of PEDOT: PSS was deposited by spin-coating a PEDOT: PSS solution (Al 4083) at 4000 rpm and dried subsequently at 150 °C for 20 min in air. Then the substrates were transferred to a nitrogen filled glove box, where the polymer donor and the acceptor blend layer was spin-coated using a solution of polymer donor : acceptor blend onto

the PEDOT: PSS layer via different spin speed. The substrates were then placed on the hotplate, annealed at different temperatures for 10 min in nitrogen. Finally, a thin layer of LiF (1 nm) and a layer of Al (100 nm) electrode were deposited in vacuum on the substrate at a pressure of ca. 5.0 × 10^{-6} Pa. The active area of the devices is 0.0574 cm^2. The current density–voltage (J-V) characteristics of the polymer solar cells were measured on an Agilent B2912A Semiconductor Analyser with a ScienceTech SLB300-A Solar Simulator. A 450 W xenon lamp and an air mass (AM) 1.5 filter were used as the light source.

4. Synthesis

4.1. Synthesis of 5-bromothiophene-3-carboxylic acid

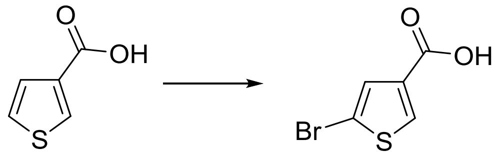

To a solution of thiophene-3-carboxylic acid (6.00 g, 46.8mmol) in 60 mL of acetic acid, bromine (7.47 g, 46.8 mmol) was added dropwise. The mixture was stirred at room temperature overnight and then water was added. The white precipitates were filtered and recrystallized with a warm mixture of water: methanol (4:1) to give the desired compound as white solid. Yield: 4.68 g (49%) ^{1}H NMR (300 MHz, $CDCl_3$): δ 8.11 (d, *J* = 1.5 Hz, 1H), 7.51 (d, *J* = 1.5 Hz, 1H). ^{13}C NMR (75 MHz, $CDCl_3$): δ 167.3, 136.2, 133.4, 130.7, 113.7.

4.2. Synthesis of 5-bromo-N,N-dibutylthiophene-3-carboxamide

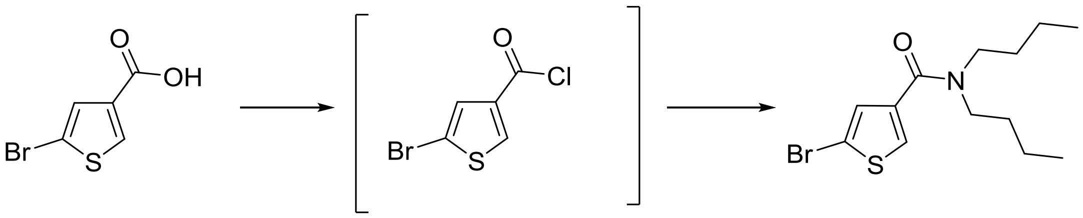

To a solution of 5-bromothiophene-3-carboxylic acid (1.64 g, 7.9 mmol) in 12 mL of

anhydrous chloroform in a 50 ml heat gun-dried two-neck round bottom flask. The solution was cooled to 0 °C in an ice bath. Oxalyl chloride (2.00 g, 15.8 mmol) was added dropwise followed with one drop of DMF as a catalyst. The solution was allowed to warm to room temperature and stirred for 4 h. The unreacted excess oxalyl chloride was removed under reduced pressure. This intermediate product was used immediately for the next step without further purification.

To a 50 ml heat gun-dried two-neck round bottom flask, dibutylamine (2.05 g, 15.8 mmol) in anhydrous chloroform (10 mL) was added. The solution was cooled to 0 °C in an ice bath. The crude 5-bromothiophene-3-carbonyl chloride synthesized above was dissolved in anhydrous chloroform (5 mL) was added dropwise. Then the solution was allowed to warm to room temperature and stirred overnight. The reaction was quenched by addition of water. The organic phase was washed with brine twice and dried over anhydrous Na_2SO_4. Upon removal of solvent in vacuo, the crude product was further purified by column chromatography on silica gel with hexane: ethyl acetate (5:1) to give the target compound. Yield: 2.33 g (95%). ^{1}H NMR (300 MHz, $CDCl_3$): δ 7.31 (s, 1H), 7.10 (s, 1H), 3.34 (d, *J* = 26.8 Hz, 4H), 1.64 – 1.13 (m, 8H), 0.90 (m, 6H). ^{13}C NMR (75 MHz, $CDCl_3$): δ 167.3, 136.2, 133.4, 130.7, 113.7.

4.3. Synthesis of $N^4,N^4,N^{4'},N^{4'}$-tetrabutyl-[2,2'-bithiophene]-4,4'-dicarboxamide

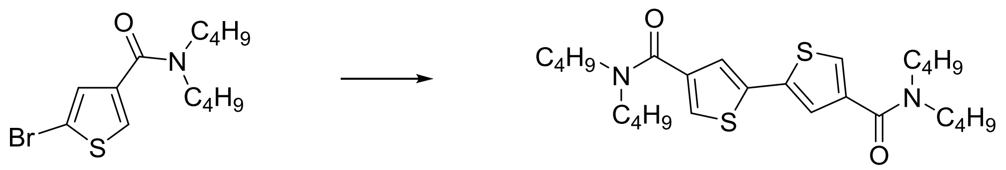

To a 50 mL round bottom flask, 5-bromo-N,N-dibutylthiophene-3-carboxamide (2.23 g, 7 mmol), zinc powder (1.83 g, 28 mmol), triphenylphosphine (0.92 g, 3.5 mmol), 2,2’-bipyridyl (0.077 g, 0.49 mmol), and anhydrous nickel (II) chloride (0.063 g, 0.49 mmol) were added. The mixture was vacuumed and filled with argon three times and then DMAc (15 mL) was added. The mixture was heated to 80 °C and stirred overnight (the TLC showed no starting material left). The reaction mixture was cooled down to room temperature and poured into a cold dilute HCl solution. After extraction with ethyl acetate, the organic phase was dried with anhydrous Na_2SO_4. The solvent was removed under reduced pressure. Purification via column chromatography on silica gel with

hexane: ethyl acetate (3:1) gave the desired product. Yield: 1.05 g (63%). ^{1}H NMR (300 MHz, $CDCl_3$): δ 7.31 (d, *J* = 1.3 Hz, 2H), 7.22 (d, *J* = 1.3 Hz, 2H), 3.39 (d, *J* = 28.5 Hz, 8H), 1.62 (s, 24H), 0.92 (d, *J* = 21.4 Hz, 12H). ^{13}C NMR (75 MHz, $CDCl_3$): δ 167.3, 136.2, 133.4, 130.7, 113.7.

4.4. Synthesis of 5,5'-dibromo-N^4,N^4,$N^{4'}$,$N^{4'}$-tetrabutyl-[2,2'-bithiophene]-4,4'-dicarboxamide

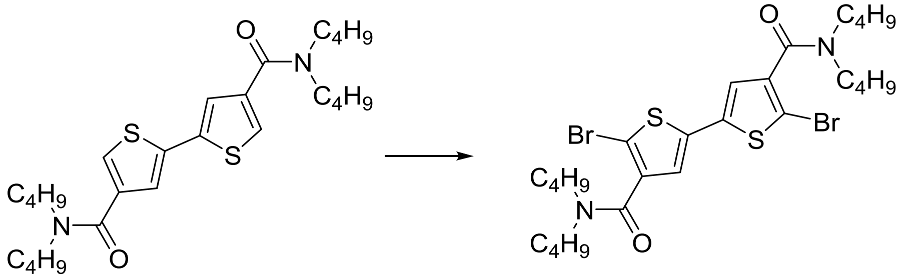

To a 25ml two-neck round bottom flask, N^4,N^4,$N^{4'}$,$N^{4'}$-tetrahexyl-[2,2'-bithiophene]-4,4'-dicarboxamide (0.74 g, 1.5 mmol) and NBS (0.58 g, 3.15 mmol) were added. The mixture was vacuumed and filled with argon three times and then a mixture of chloroform (7.5 mL) and trifluoroacetic acid (1.5 mL) were added. The mixture was stirred in the dark for 5 h (TLC showed no starting material left). The mixture was extracted with chloroform several times and the organic phase were combined and washed with sodium sulfite aqueous solution. The organic phase was dried with anhydrous Na_2SO_4 and the solvent was removed under reduced pressure. Purification via column chromatography on silica gel with ethyl acetate/hexane (1:3) gave the desired product. Yield: 0.90 g (94%). ^{1}H NMR (300 MHz, $CDCl_3$): δ 6.89 (s, 2H), 3.54 – 3.42 (t, *J* = 7.5 Hz, 4H), 3.22 (t, *J* = 7.5 Hz, 4H), 1.72 – 1.33 (m, 16H), 0.97 (t, *J* = 7.3 Hz, 6H), 0.82 (t, *J* = 7.3 Hz, 6H). ^{13}C NMR (75 MHz, $CDCl_3$): δ 167.3, 136.2, 133.4, 130.7, 113.7.

4.5. Synthesis of 5-bromo-N,N-dihexylthiophene-3-carboxamide

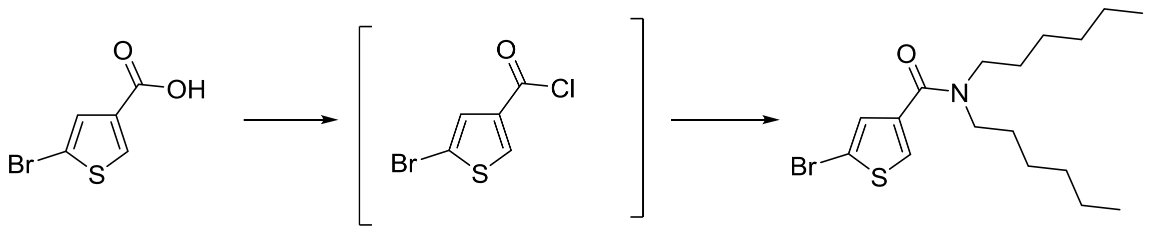

The solution of (1) 5-bromothiophene-3-carboxylic acid (1.86 g, 9 mmol) in 12 mL of anhydrous chloroform was put in a 50 mL heat gun-dried two-neck round bottom flask. The solution was cooled to 0 °C in an ice bath. Oxalyl chloride (2.29 g, 18 mmol) was added dropwise followed with one drop of dimethylformamide (DMF) as a catalyst. The solution was allowed to warm to room temperature for 4h. Unreacted oxalyl chloride was removed under reduced pressure. The intermediate product was used immediately for the next step, due to its very poor stability in air. To a 50 mL heat gun-dried two-neck round bottom flask, dihexylamine (3.34 g, 15 mmol) in 10 mL of anhydrous chloroform was added. The solution was cooled to 0 °C in an ice bath. Crude 5-bromothiophene-3-carbonyl chloride was dissolved in 5 mL of anhydrous chloroform and added dropwise carefully. Then the solution was allowed to warm to room temperature and stirred overnight. The reaction was quenched by addition of water. The organic phase was further washed with brine twice and dried over anhydrous Na_2SO_4. Upon removal of solvent in vacuo, the crude product was further purified by silica gel column chromatography with hexane: ethyl acetate (5:1) to give Compound 2. Yield: 3.23 g, (96%).

^{1}H NMR (300 MHz, $CDCl_3$): δ 7.32 (s, 1H), 7.10 (s, 1H), 3.34 (d, *J* = 31.0 Hz, 4H), 1.42 (dd, *J* = 99.2, 21.6 Hz, 18H), 0.89 (d, *J* = 5.8 Hz, 6H).

4.6. Synthesis of N4,N4,N4',N4'-tetrahexyl-[2,2'-bithiophene]-4,4'-dicarboxamide

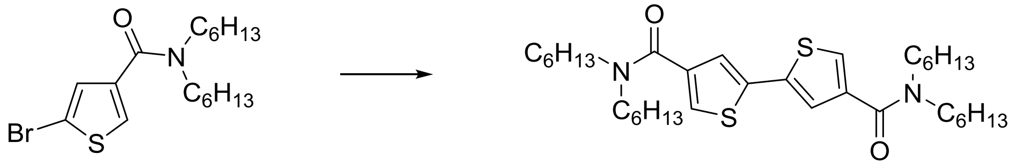

To a 50 mL round bottom flask, N, N-dihexyl-3-thiophenecarbamide (2.6 g, 7 mmol), zinc powder (1.83 g, 28 mmol), triphenylphosphine (0.92 g, 3.5 mmol), 2,2'-bipyridyl (0.077 g, 0.49 mmol), anhydrous nickel (II) chloride (0.063 g, 0.49 mmol) were added. The mixture was vacuumed and filled with argon three times and then dimethylacetamide (DMAc) (20 mL) was added. The mixture was heated to 80 °C and maintained overnight (the thin layer chromatography (TLC) showing no starting material). The reaction mixture was cooled down to room temperature and poured into a cold HCl. After extraction with ethyl acetate, the organic phase was dried with

anhydrous Na_2SO_4. The solvent was removed under reduced pressure. Purification via silica gel column chromatography with hexane: ethyl acetate (3:1) to give Compound 3. Yield: 1.20 g, (57%).

^{1}H NMR (300 MHz, $CDCl_3$): δ 7.31 (s, 2H), 7.22 (s, 2H), 3.38 (d, *J* = 27.5 Hz, 8H), 1.54 (m, 8H), 1.32 (m, 24H), 0.88 (s, 12H).

4.7. Synthesis of 5,5'-dibromo-N4,N4,N4',N4'-tetrahexyl-[2,2'-bithiophene]-4,4'-dicarboxamide

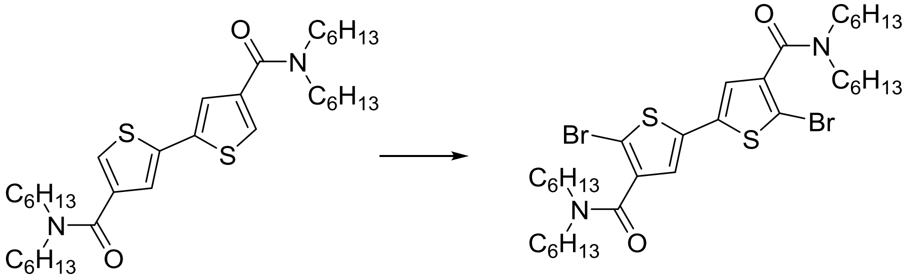

To a 25 mL two-neck round bottom flask, N^4, N^4, $N^{4'}$, $N^{4'}$-tetrahexyl-[2,2'-bithiophene]-4,4'-dicarboxamide (3) (0.59 g, 1.0 mmol) and N-bromosuccinimide (NBS) (0.38 g, 2.1 mmol) were added. The mixture was degassed and filled with argon three times and then mixture of chloroform (5 mL) and trifluoroacetic acid (1 mL) were added. The mixture was stirred in the dark covered with aluminum foil for 2 h (TLC showed no starting material left). The mixture was extracted with chloroform several times and the organic phases were combined washed by sodium sulfite aqueous solution. The organic phase was dried with anhydrous Na_2SO_4 and the solvent was removed under reduced pressure. Purification via column chromatography on silica gel with ethyl acetate/hexane (1:4) gave the desired product. Yield: 0.75 g (95%).

^{1}H NMR (300 MHz, $CDCl_3$): δ 6.89 (s, 2H), 3.47 (t, *J* = 7.6 Hz, 4H), 3.22 (t, *J* = 7.5 Hz, 4H), 1.65 – 1.49 (m, 8H), 1.34 – 1.15 (m, 24H), 0.87 (d, *J* = 21.4 Hz, 12H). ^{13}C NMR (75 MHz, $CDCl_3$): δ 164.61, 139.04, 137.02, 123.63, 109.49, 48.45, 44.65, 31.56, 31.18, 28.56, 27.35, 26.66, 26.16, 22.58, 22.41, 14.02, 13.94.

4.8. Synthesis of Polymer 66BT

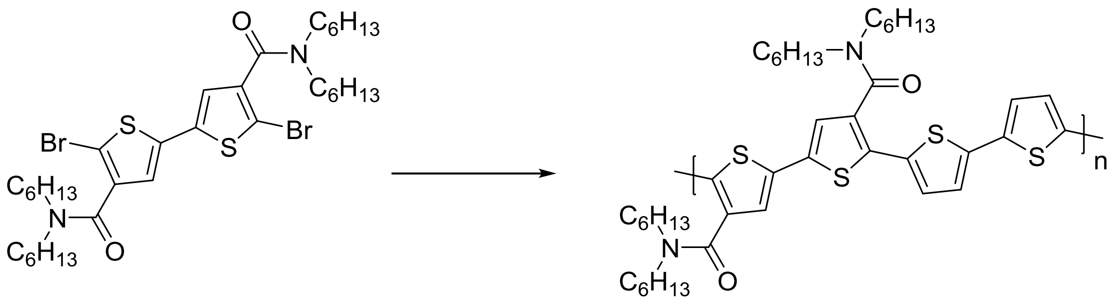

To a 25 mL double-neck round-bottom flask, 5,5'-dibromo-N[4], N[4], N[4'], N[4']-tetrahexyl-[2,2'-bithiophene]-4,4'-dicarboxamide (153 mg, 0.205 mmol) and 5, 5'-bis(trimethylstannyl)-2,2'-bithiophene (100 mg, 0.205 mmol) were added. After degassing and refilling argon three times, toluene (4 mL) was added to dissolve the mixture, followed by addition of $Pd(PPh_3)_4$ (12 mg, 0.01 mmol). The mixture was heated to reflux for 24 h. The reaction mixture was cooled down to room temperature and poured into MeOH (150 mL). The precipitates were collected by filtration and subjected to Soxhlet extraction with acetone and hexane successively. The residual was dissolved with chloroform. The polymer was recovered as solid from the chloroform fraction by precipitation from methanol. Yield: 128 mg, (80.0 %) in chloroform fraction. GPC data: M_w: 58.4 kDa, M_n: 23.4 kDa and *Đ*: 2.5.

4.9. Synthesis of Polymer 44BT

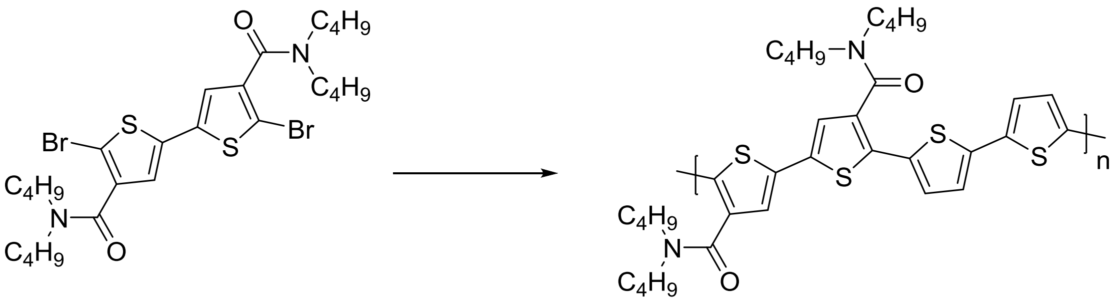

To a 25 mL double-neck round-bottom flask, 5,5'-dibromo-N4, N4, N4', N4'-tetrabutyl-[2,2'-bithiophene]-4,4'-dicarboxamide (190 mg, 0.3 mmol) and 5,5'-bis(trimethylstannyl)-2,2'-bithiophene (148 mg, 0.3 mmol) were added. After degassing and refilling argon three times, toluene (5 mL) was added to dissolve the mixture, followed by addition of $Pd(PPh_3)_4$ (17 mg, 0.015

mmol). The mixture was heated to reflux for 24 h. The reaction mixture was cooled down to room temperature and poured into MeOH (150 mL). The precipitate was collected by filtration and subjected to Soxhlet extraction with acetone and hexane successively. The residual was dissolved in chloroform. The polymer was recovered as solid from the chloroform fraction by precipitation from methanol. The solid was dried under vacuum. Yield: 155 mg, (78.0%). MALDI-TOF-MS: M_w: 7.2 kDa, M_n: 6.5 kDa and $Đ$: 1.1.

4.10. Synthesis of Polymer 44TT

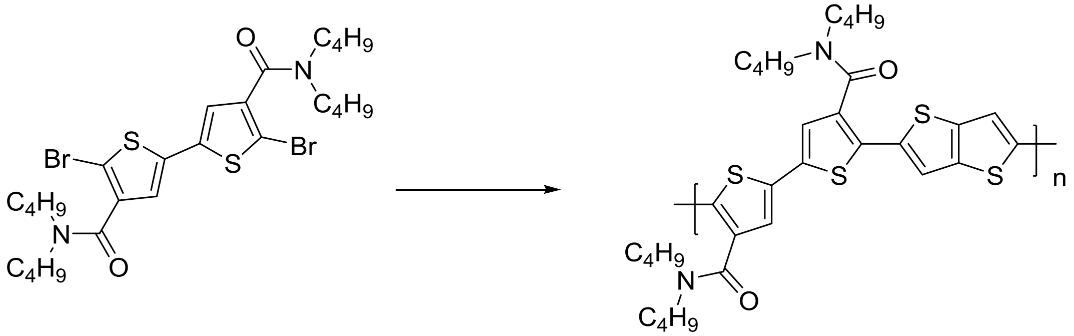

To a 25 ml double-neck round-bottom flask 5,5'-dibromo-N4,N4,N4',N4'-tetrabutyl-[2,2'-bithiophene]-4,4'-dicarboxamide (159 mg, 0.25 mmol) and 2,5-bis(trimethylstannyl)-thieno[3,2-b]thiophene (116 mg, 0.25 mmol). After degassing and refilling argon three times, chlorobenzene (4 mL) was added to dissolve the mixture, followed by an addition of $Pd(PPh_3)_4$ (14 mg, 0.013 mmol). The reaction mixture was heated to 120 ℃ and stirred for 40 h. The reaction mixture was cooled down to room temperature and poured into MeOH (100 ml). The precipitates were collected by filtration and subjected to Soxhlet extraction with acetone, hexane and chloroform successively. The residual was dissolved in chlorobenzene. The polymer was recovered as solid from the chlorobenzene fraction by precipitation from methanol. Yield: 109 mg (78.0%). MALDI-TOF-MS: M_w: 8.47 kDa, M_n: 7.7 kDa and $Đ$: 1.1.

5. Additional data

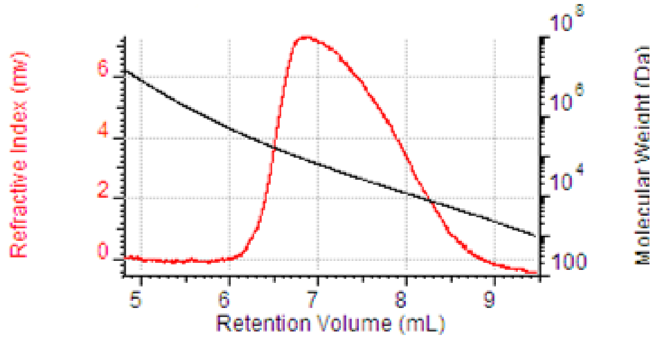

Figure S1. The molecular weight distribution of 66BT obtained by HT-GPC at 140 °C with 1,2,4-trichlorobenzene as eluent and polystyrene standards.

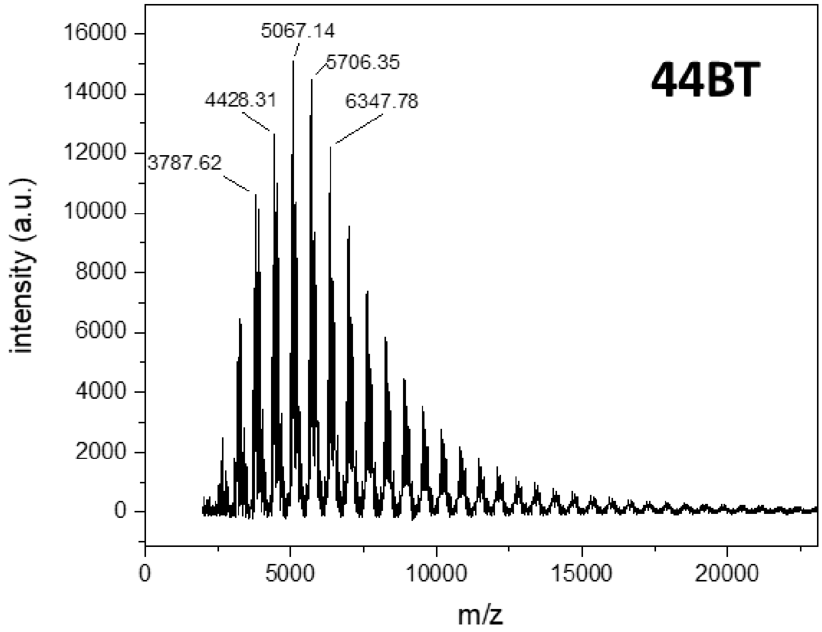

Figure S2. The molecular weight distribution of 44BT obtained by MALDI-TOF-MS from a matrix of DCTB (2500:1 matrix-to-polymer ratio) casted from chloroform.

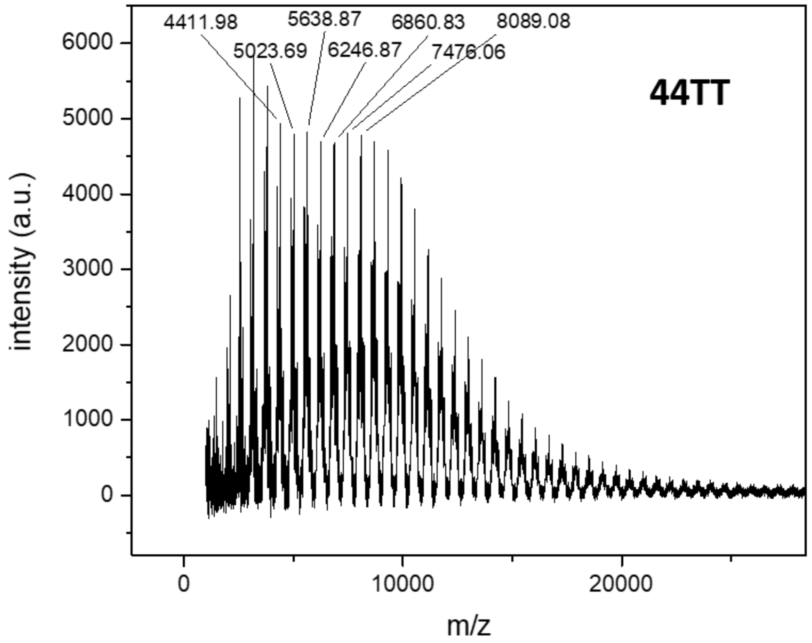

Figure S3. The molecular weight distribution of 44TT obtained by MALDI-TOF-MS from a matrix of DCTB (2500:1 matrix-to-polymer ratio) casted from chloroform.

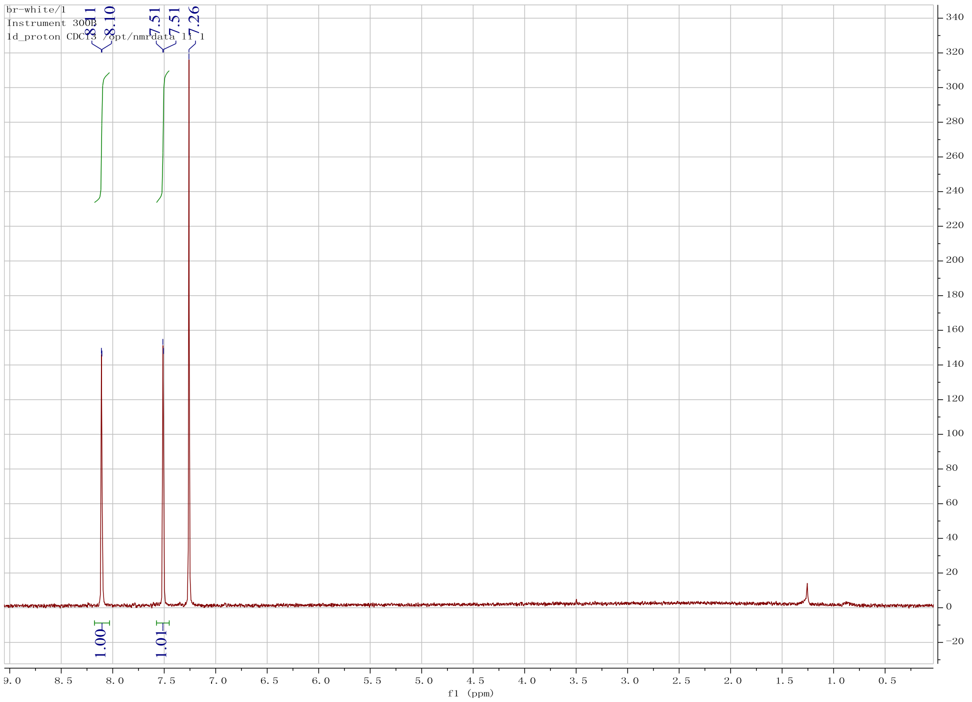

Figure S4. 300 MHz ^{1}H NMR spectrum for 5-Bromothiophene-3-carboxylic acid in Chloroform-*d*

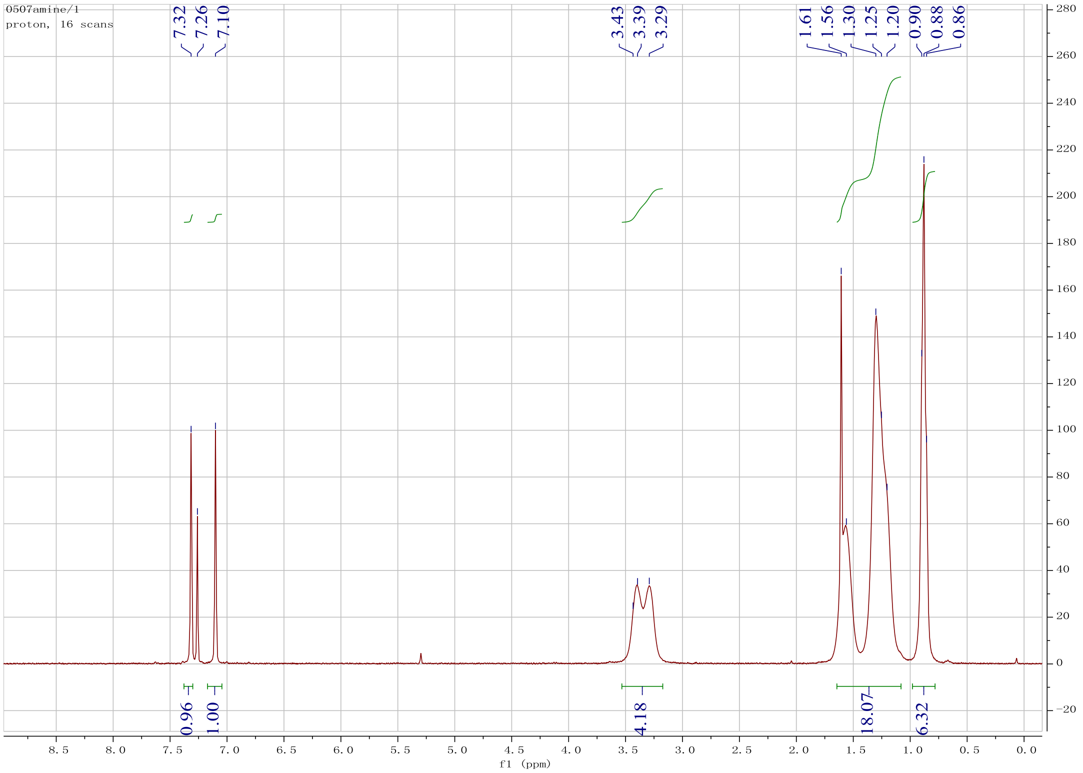

Figure S5. 300 MHz ^{1}H NMR spectrum for N, N-dihexyl-3-thiophenecarbamide in chloroform-*d*.

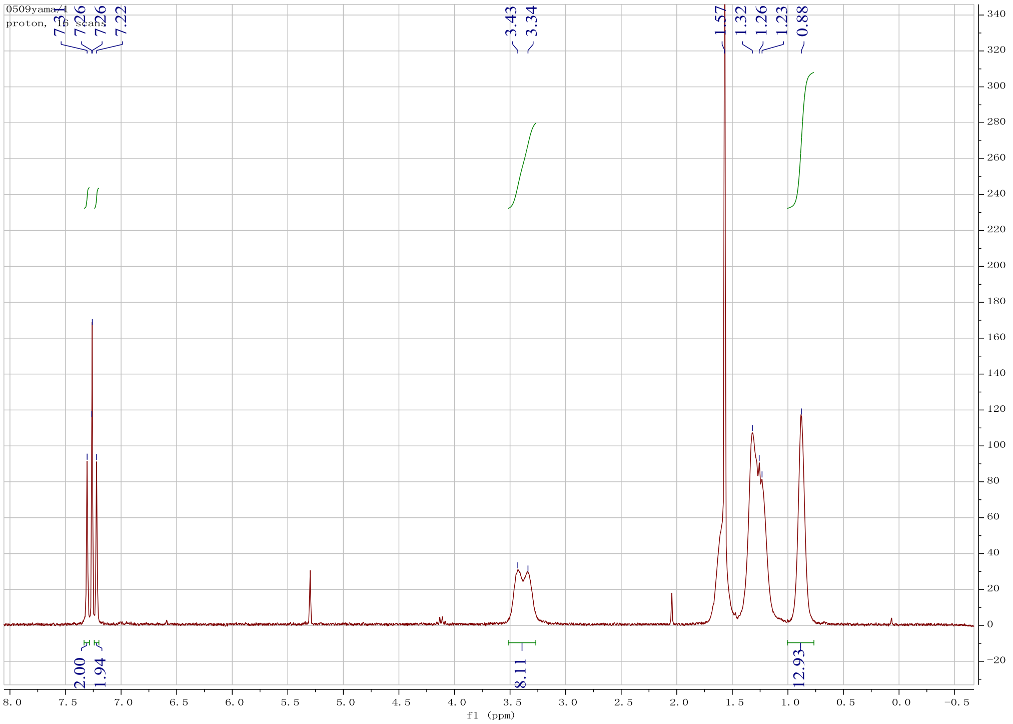

Figure S6. 300 MHz ^{1}H NMR spectrum for N^{4}, N^{4}, N$^{4'}$, N$^{4'}$-tetrahexyl-[2,2'-bithiophene]-4,4'-dicarboxamide in chloroform-*d*.

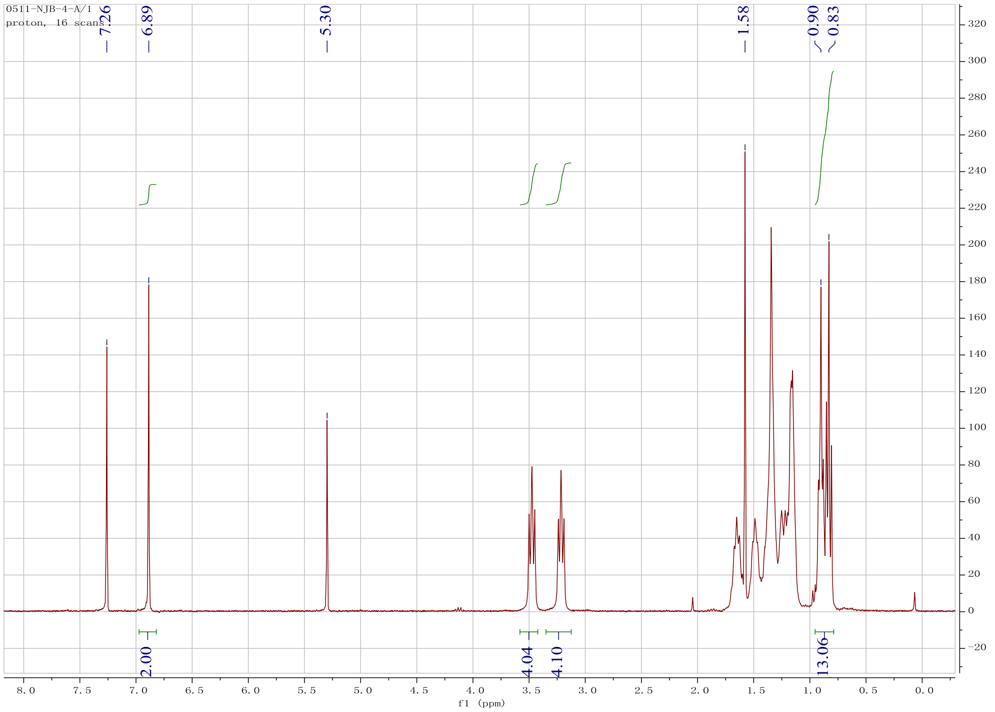

Figure S7. 300 MHz ^{1}H NMR spectrum for 5,5'-dibromo-N^{4}, N^{4}, N$^{4'}$, N$^{4'}$-tetrahexyl-[2,2'-bithiophene]-4,4'-dicarboxamide in chloroform-*d*.

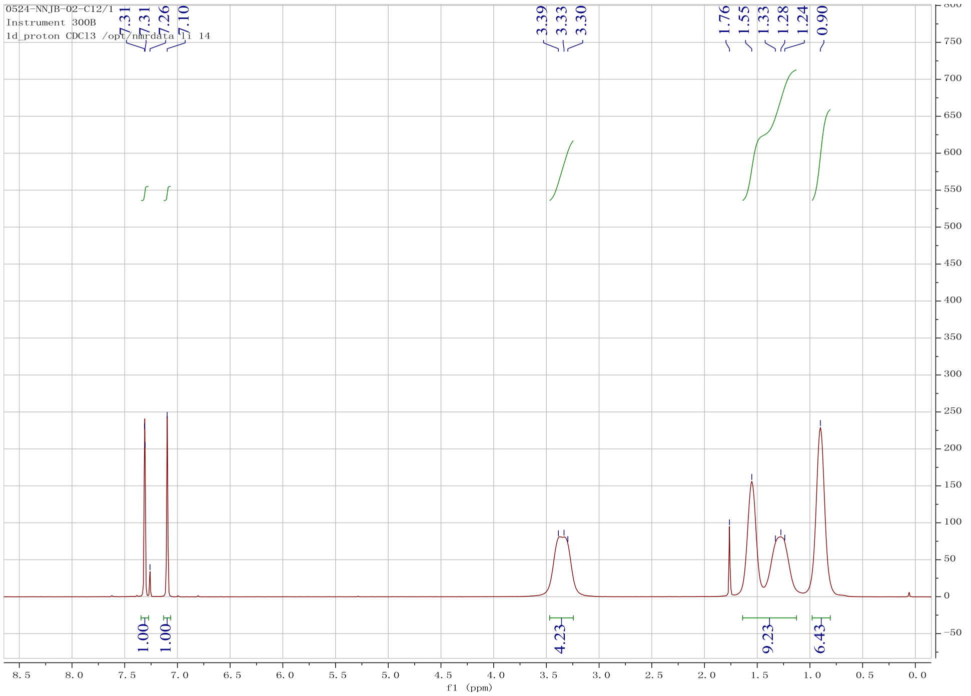

Figure S8. 300 MHz ^{1}H NMR spectrum for 5-bromo-N,N-dibutylthiophene-3-carboxamide in Chloroform-*d*

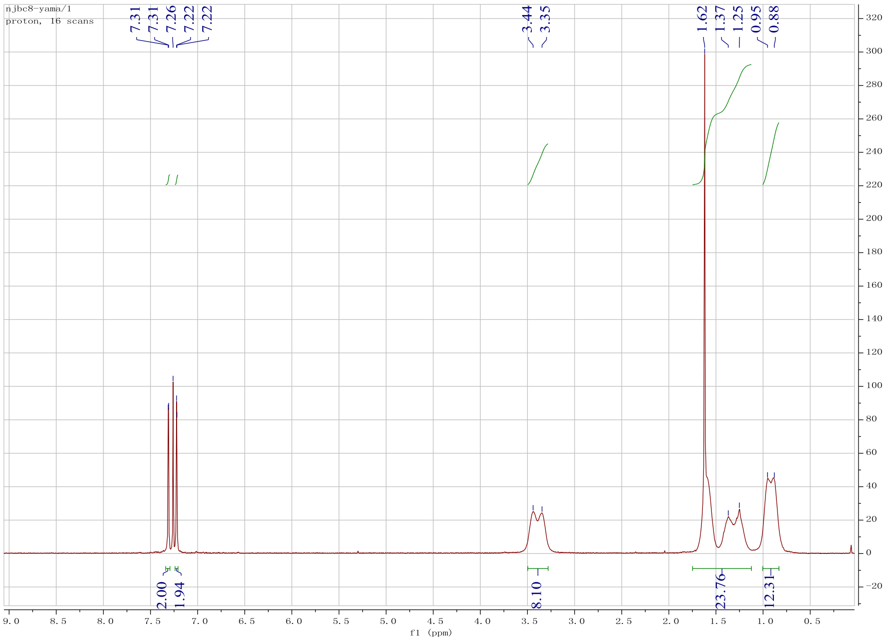

Figure S9. 300 MHz ^{1}H NMR spectrum for N^{4},N^{4},$N^{4'}$,$N^{4'}$-tetrabutyl-[2,2'-bithiophene]-4,4'-dicarboxamide in Chloroform-*d*

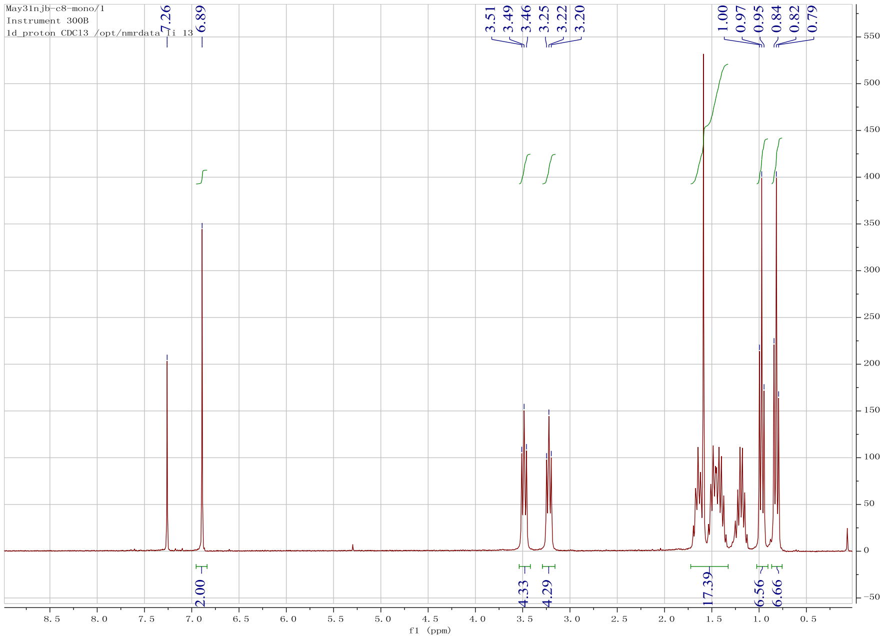

Figure S10. 300 MHz ^{1}H NMR spectrum for 5,5'-dibromo-N^4,N^4,N$^{4'}$,N$^{4'}$-tetrabutyl-[2,2'-bithiophene]-4,4'-dicarboxamide in Chloroform-*d*

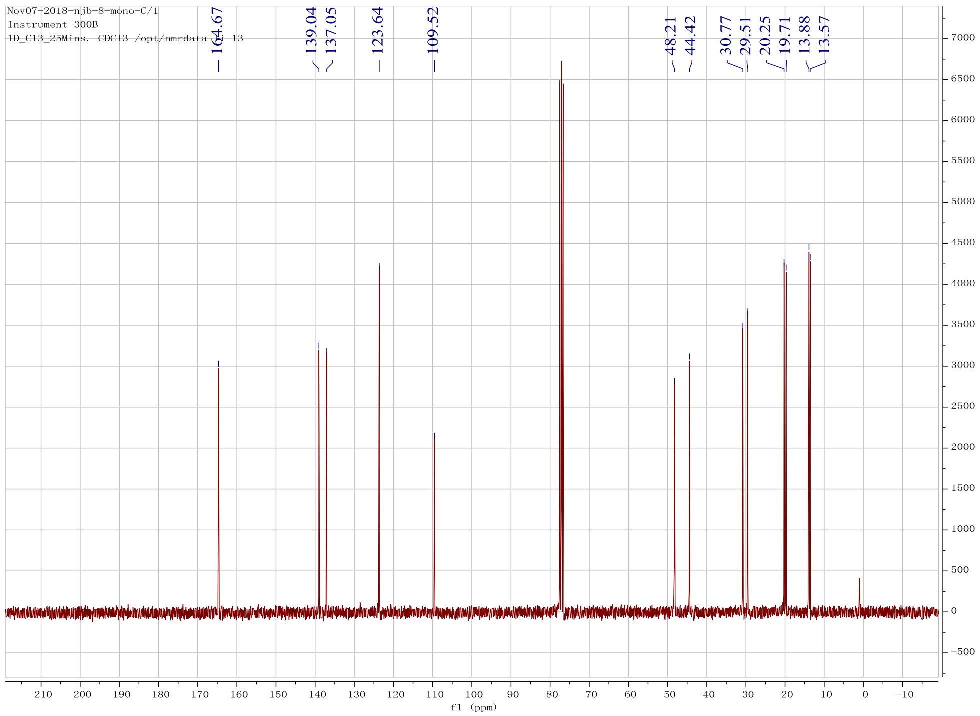

Figure S11. 75 MHz ^{13}C NMR spectrum for 5,5'-dibromo-N^4, N^4, N$^{4'}$, N$^{4'}$-tetrahexyl-[2,2'-bithiophene]-4,4'-dicarboxamide in chloroform-*d.*

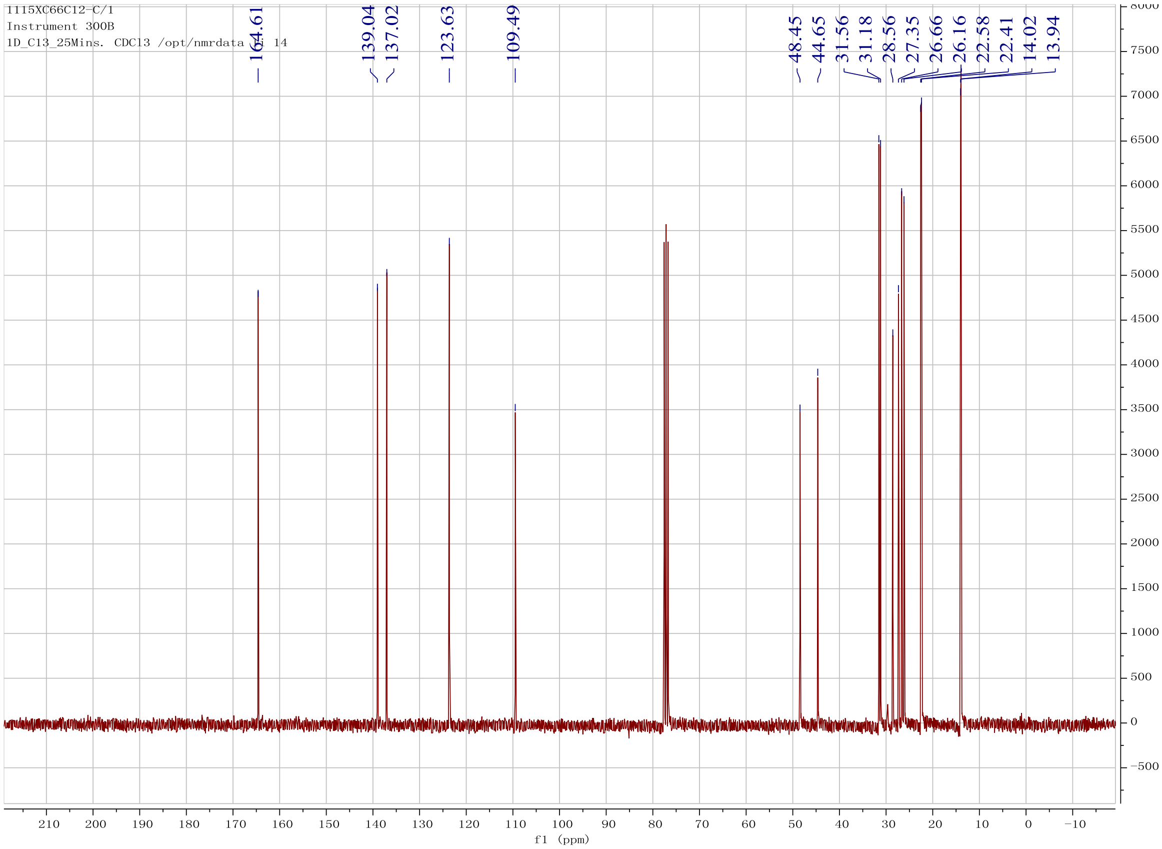

Figure S12 .75 MHz ^{13}C NMR spectrum for 5,5'-dibromo-N^{4}, N^{4}, N$^{4'}$, N$^{4'}$-tetrabutyl-[2,2'-bithiophene]-4,4'-dicarboxamide in chloroform-*d.*

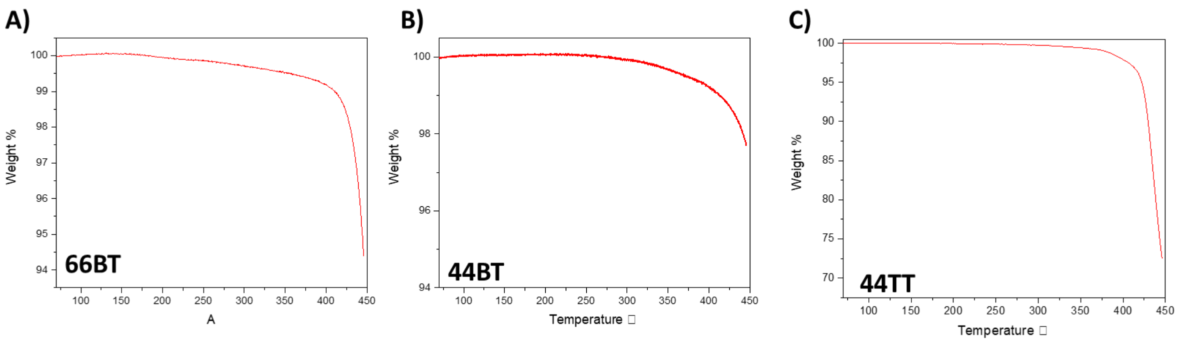

Figure S13._The TGA curves of A) 66BT B) 44BT and C) 44TT with increasing rate of 10 °C min^{-1} obtained in nitrogen.

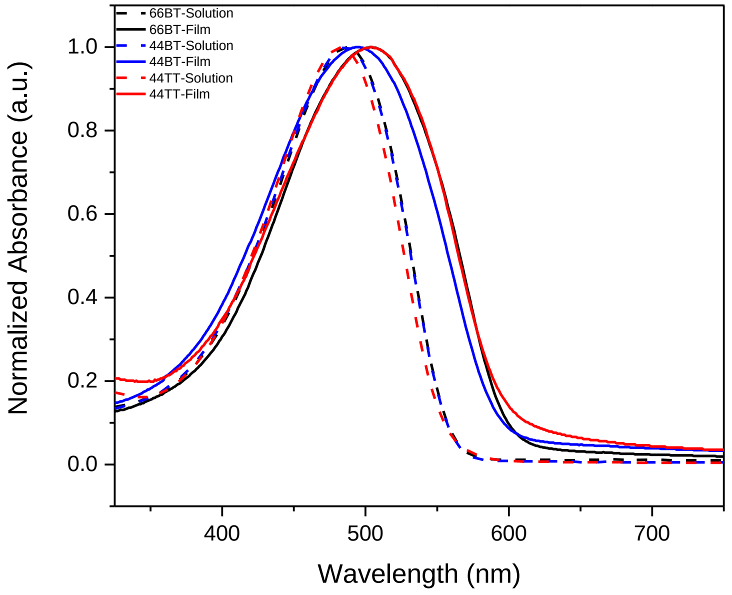

Figure S14. UV-Vis absorption spectra of 66BT, 44BT, and 44TT in chloroform solutions and as thin films.

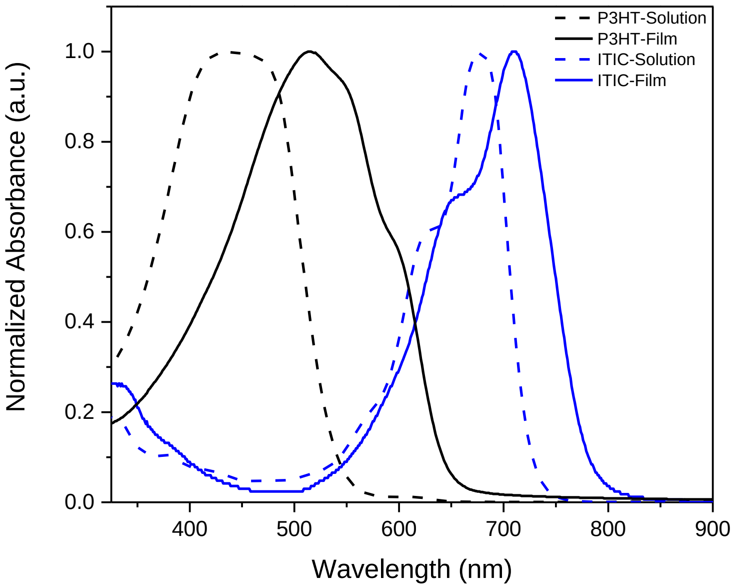

Figure S15. UV-Vis absorption spectra of P3HT and ITIC in chloroform solutions and as thin films.

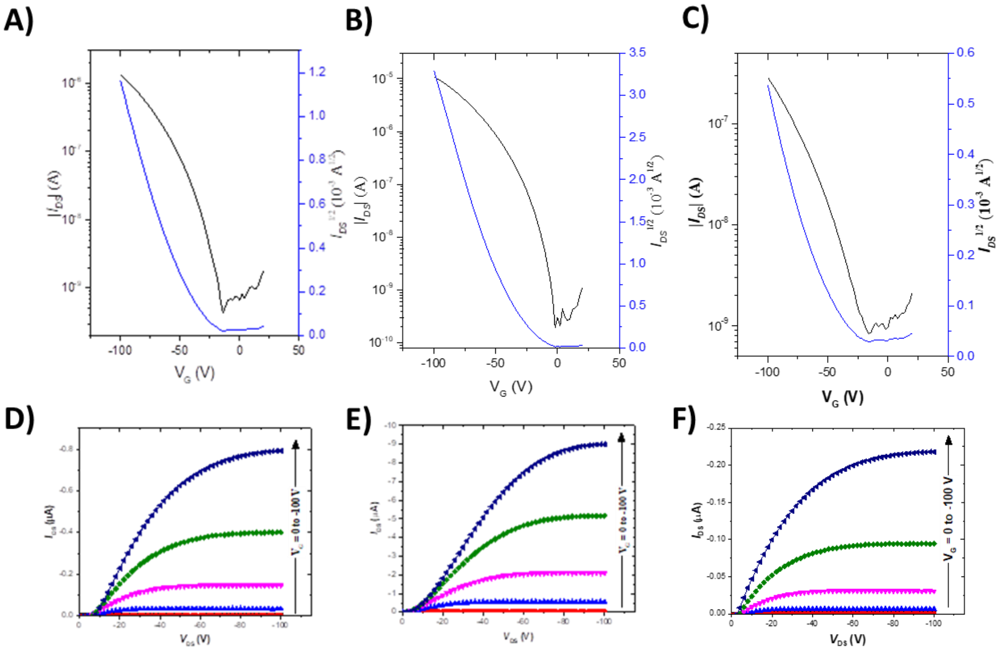

Figure S16. The transfer curves for OTFTs based on (A) 66BT (150 °C), (B) 44BT (200 °C), (C) 44TT (200 °C) and output curves for OTFTs based on (D) 66BT (150 °C), (E) 44BT (200 °C) and (F) 44TT (200 °C). Device dimensions: channel length (L) = 30 µm; channel width (W) = 1000 µm. V_G valued from 0 to -100 V in the step of -20 V in output curves.

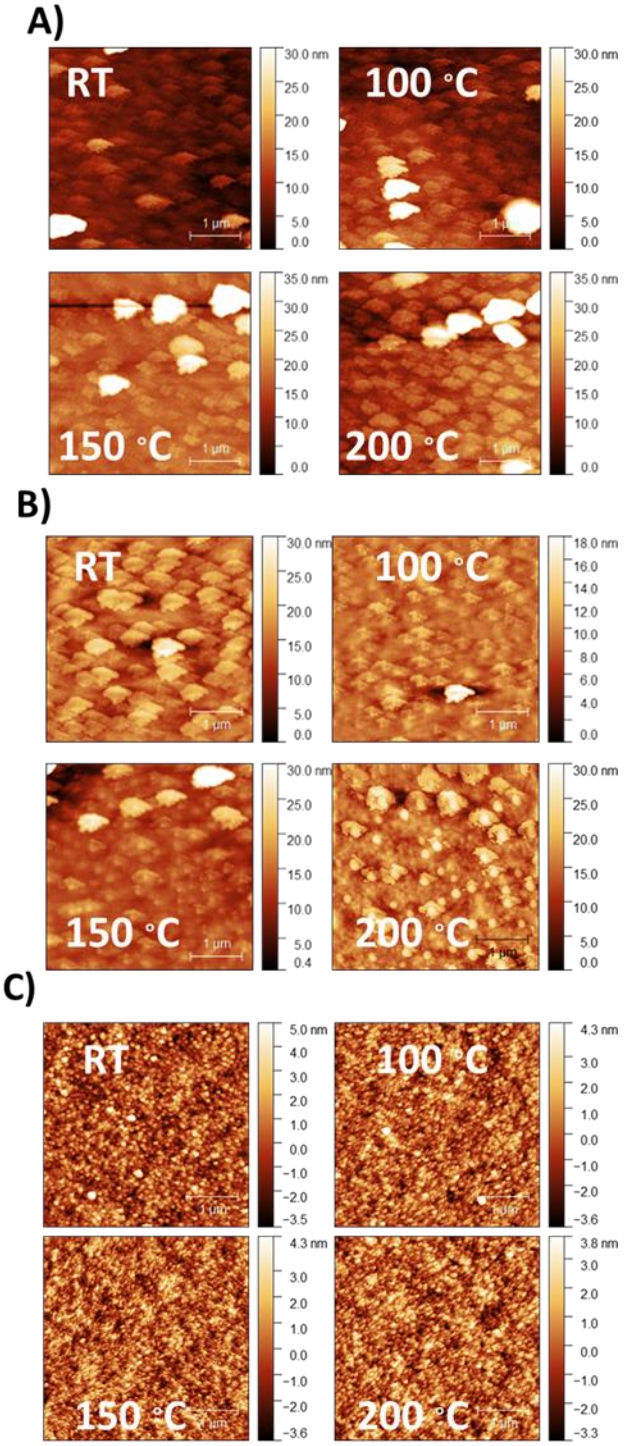

Figure S17 AFM height images (4 µm× 4 µm) of A) 66BT B) 44BT and C) 44TT thin films spin-coated on DDTS-modified SiO_2/Si substrates and annealed at different temperatures for 20 min under nitrogen.

Table S1 OTFT device parameters of 66BT, 44BT and 44TT at different annealing temperatures.

Polymer	Annealing[a] temperature (°C)	Hole mobility[b] (10^{-3} cm^2 V^{-1} s^{-1})	V_{th}[c] (V)	$I_{ON/OFF}$
66BT	RT	0.19 ± 0.01 (0.21)	-53	10^3
	100	1.0 ± 0.08 (1.1)	-56	10^3
	150	1.7 ± 0.23 (2.0)	-37	10^3
	200	0.3 ± 0.5 (0.4)	-56	10^4
44BT	RT	0.29 ± 0.03 (0.33)	-37	10^3
	100	5.8 ± 0.87 (6.7)	-38	10^4
	150	11 ± 1.7 (13)	-38	10^4
	200	14 ± 1.0 (14)	-41	10^5
44TT	RT	0.71 ± 0.042 (0.75)	-27	10^3
	100	0.93 ± 0.020 (0.94)	-33	10^3
	150	0.98 ± 0.010 (1.0)	-38	10^3
	200	4.2 ± 0.26 (4.5)	-44	10^3

[a] The devices were annealed in a glove box on a hotplate at the selected temperature for 20 min under argon. Hole mobilities were obtained in the saturated regions in hole enhancement modes. Each set of data were obtained from 3-5 OTFT devices.

[b] The average mobility ± standard deviation (maximum mobility) calculated from the saturation regions of the devices.

[c] The V_{th} calculated from the device with maximum mobility.

Table S2 Solar cell data for 44BT, 66BT and 44TT.

Active layer	Ratio	Solvent	Annealing temperature (℃)	Jsc (mA/cm²)	Voc (V)	FF	PCE %	Rs (ohm/ cm²)	Rsh (ohm/ cm²)
66BT: PCBM	1: 1	CB	RT	2.7	0.87	0.31	0.72	319	399
66BT: PCBM	1: 1	CB	120 ℃	1.6	0.74	0.25	0.31	430	262
44BT: PCBM	1: 1	CB	RT	2.8	0.85	0.34	0.80	149	529
44BT: PCBM	1: 1	CB	120 ℃	2.4	0.72	0.29	0.50	187	424
66BT: PCBM	1: 1	DCB	RT	2.7	0.86	0.31	0.70	360	457
66BT: PCBM	1: 1	DCB	120 ℃	2.5	0.75	0.30	0.55	260	481
66BT: PCBM	1: 1	DCB	RT	4.2	0.87	0.36	1.31	87	353
66BT: PCBM	1: 1	DCB	120 ℃	1.6	0.71	0.29	0.33	186	452
66BT: PCBM	1.5: 1	DCB	RT	2.8	0.87	0.30	0.73	340	532
66BT: PCBM	1: 1	DCB	RT	4.2	0.87	0.36	1.31	87	353
66BT: PCBM	1: 1.5	DCB	RT	2.8	0.78	0.34	0.73	268	583
66BT: ITIC	1: 1	DCB	RT	3.5	0.81	0.30	0.85	269	349

44TT: PCBM	1: 1	CB	RT	4.8	0.82	0.41	1.59	87	420
44TT: PCBM	1: 1	DCB	RT	3.5	0.82	0.38	1.08	363	590
44TT: ITIC	1: 1	CB	RT	7.3	0.89	0.32	2.04	48	166
44TT: ITIC	1: 1	DCB	RT	11.25	0.88	0.38	3.70	90	226
44TT: ITIC	1:1	DCB + DIO (0.5% v/v)	RT	8.2	0.92	0.35	2.68	67	253
44TT: ITIC	1:1	DCB [a]	RT	8.3	0.91	0.43	3.29	134	392
44TT: ITIC	1:1	DCB [a]	RT (warm substrate)	13.22	0.87	0.39	4.47	85	309
P3HT: ITIC	1:1	CF	160 ℃	3.33	0.52	0.53	0.92	39	935

[a] The total concentration of D/A blend is 16 mg ml^{-1}. For other systems, the total concentration is 20 mg ml^{-1} .
